# Long-Term Climate Forcing in Loggerhead Sea Turtle Nesting

**DOI:** 10.1371/journal.pone.0019043

**Published:** 2011-04-27

**Authors:** Kyle S. Van Houtan, John M. Halley

**Affiliations:** 1 Marine Turtle Assessment Program, NOAA Fisheries Service, Pacific Islands Fisheries Science Center, Honolulu, Hawaii, United States of America; 2 Nicholas School of the Environment and Earth Sciences, Duke University, Durham, North Carolina, United States of America; 3 Department of Biological Applications and Technology, University of Ioannina, Ioannina, Greece; Institut Pluridisciplinaire Hubert Curien, France

## Abstract

The long-term variability of marine turtle populations remains poorly understood, limiting science and management. Here we use basin-scale climate indices and regional surface temperatures to estimate loggerhead sea turtle (*Caretta caretta*) nesting at a variety of spatial and temporal scales. Borrowing from fisheries research, our models investigate how oceanographic processes influence juvenile recruitment and regulate population dynamics. This novel approach finds local populations in the North Pacific and Northwest Atlantic are regionally synchronized and strongly correlated to ocean conditions—such that climate models alone explain up to 88% of the observed changes over the past several decades. In addition to its performance, climate-based modeling also provides mechanistic forecasts of historical and future population changes. Hindcasts in both regions indicate climatic conditions may have been a factor in recent declines, but future forecasts are mixed. Available climatic data suggests the Pacific population will be significantly reduced by 2040, but indicates the Atlantic population may increase substantially. These results do not exonerate anthropogenic impacts, but highlight the significance of bottom-up oceanographic processes to marine organisms. Future studies should consider environmental baselines in assessments of marine turtle population variability and persistence.

## Introduction

Populations from a variety of taxa and environments have long-term correlations with climate [1], [2], [3], [4], [5]. This is particularly true in marine ecosystems where the North Atlantic Oscillation (NAO) and the Pacific Decadal Oscillation (PDO) have a dramatic influence on fisheries [6], [7]. These climate indices are correlated with population dynamics [8], [9] because they reflect atmospheric circulation patterns which regulate large scale oceanographic processes and ecosystem productivity [10], [11]. But, in contrast with the El Niño Southern Oscillation index [12], the NAO and PDO operate on decadal scales, causing extended periods of high or low population abundance [5], [7]. Though the ecological effects of these climate oscillations have been described in various settings, the influence of decadal indices to long-term marine turtle population trends is largely unexplored.

**Figure 1:**
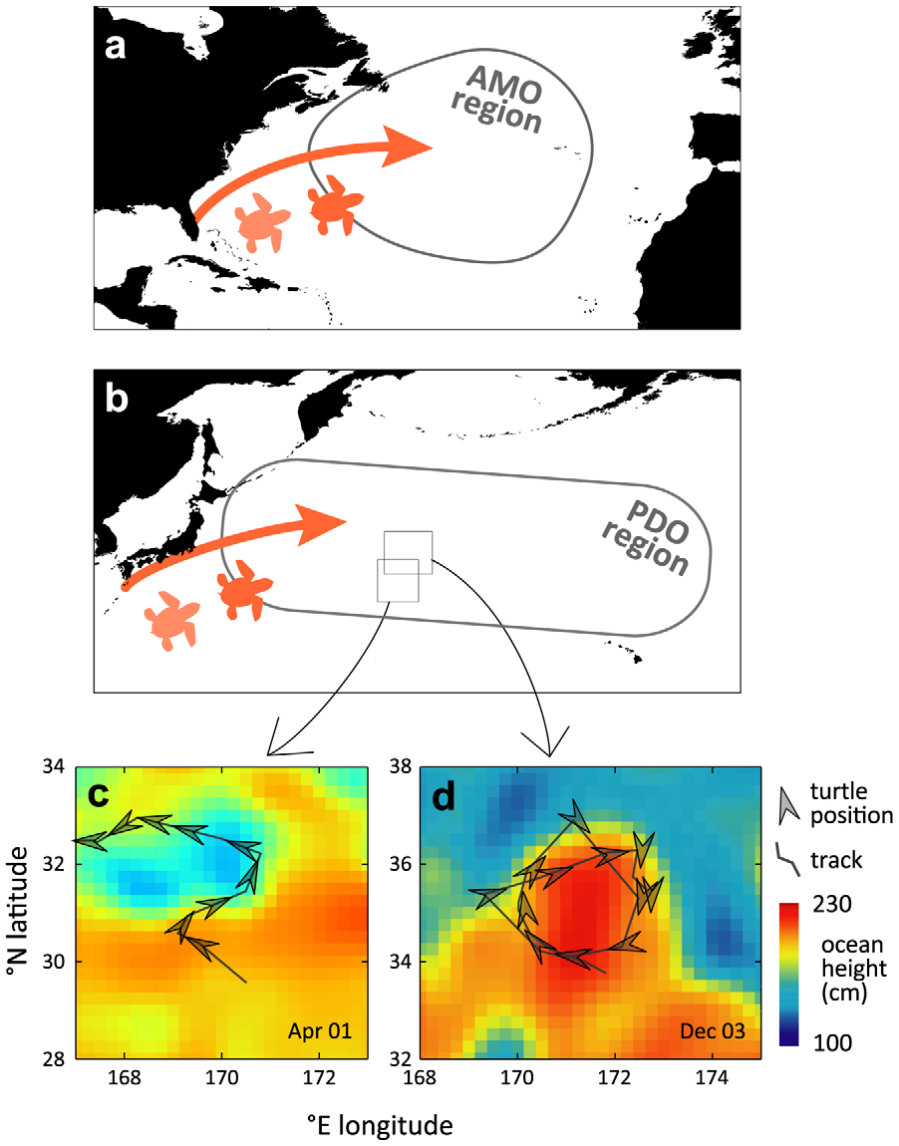
Pelagic habits of juvenile loggerhead turtles. Loggerhead juveniles disperse to regions whose climatic variability is characterized (**a**) in the North Atlantic by the Atlantic Multidecadal Oscillation (AMO) and (**b**) in the North Pacific by the Pacific Decadal Oscillation (PDO). In the North Pacific, satellite-tagged juveniles forage in cyclonic (**c**) and anti-cyclonic (**d**) feature fronts where prey are abundant. Comparable satellite studies in the Northwest Atlantic population reveal similar habits [18]. Tracking maps are redrawn from a previous study [19].

Anthropogenic pressures are considered the major driver of marine turtle populations [13], [14], [15]. A recent National Research Council (NRC) report concluded, for example, that advances in turtle population ecology will come primarily from improvements in monitoring human impacts [16]. At the same time, the NRC report noted the lack of data on juveniles to be a significant scientific challenge. After hatching loggerhead juveniles disperse to pelagic biomes (Fig 1a) thousands of kilometers from their nesting beaches where most studies occur. Although juveniles are the most numerous population segment, they are also the least accessible and least understood.

Climate indices may provide insights into the dynamics of this key demographic. Genetic [17] and tracking [18], [19] studies have revealed pelagic hotspots where juvenile loggerheads congregate; foraging in oceanographic features that are productive and concentrate prey (Fig. 1c-d). Decadal indices, as they describe the variability of these hotspots, may function as proxies for juvenile recruitment. This is the case for many marine fisheries [9]. Because juvenile population dynamics are poorly understood, this may offer immediate insights. But in addition, a climatic approach holds promise for understanding population trends - of juveniles and adults - over time.

In this paper we develop models that measure climate forcing in long-term trends of loggerhead sea turtles (*Caretta caretta*) nesting in Japan and Florida. Our models capture climate dynamics through two mechanisms: juvenile recruitment and breeding remigration. Females do not breed annually, but when ocean conditions are sufficient for females to generate yolk [20]. Nesting activity across species and ocean basins has thus been linked to sea surface temperatures (SST) in the months preceding nesting [21], [22], [23]. But where SST anomalies explain some of the annual fluctuations in nesting, recruitment variability may be the dominant driver of the long-term dynamics. Juveniles are considered more susceptible to oceanographic variability as they have a limited ability to exploit their environs for food [2], [6], [9], [24], [25]. In the North Pacific we anticipate that juvenile recruitment is correlated with the PDO, as the index is positive when atmospheric circulation is elevated [11] in the Kuroshio Bifurcation Extension Region where juveniles congregate [19] (Fig 1b–d). In the Northwest Atlantic we expect recruitment varies with the Atlantic Multidecadal Oscillation (AMO). We use this index instead of the NAO, as the AMO is strongly correlated with thermohaline and atmospheric circulation patterns [26] as well as storm activity [27], [28] in the subtropical-temperate region where Atlantic juveniles reside [17].

Our study is the first to consider how both of these important climate dynamics impact marine turtles. In doing so, a significant aspect of our models is accounting for age to maturity. Our analysis presumes juvenile recruitment dynamics can be detected when turtles are counted as breeding adults, several decades later [29]. In fisheries, age to maturity is typically a few years and correlation lags are therefore straightforward. The longest documented fisheries lag is eight years, documented both between the AMO and striped bass (*Morone saxatilis*) surveys (Bob Wood, personal communication) and between the NAO and Labrador snow crab (*Chionoecetes opilio*) landings [30]. For Northwest Atlantic loggerheads, estimates of age at first breeding range from 30–32 years [29], [31]. We therefore fix the lag in this population at 31 years. We have no maturity estimates for North Pacific loggerhead, however. We therefore model the lag of juvenile climate dynamics over a plausible range of values, allowing the models to optimize the lag distance for the Japan regional total series.

We used general linear models to estimate annual nesting at two spatial scales relevant to conservation management [31] - local and regional nesting surveys. To examine their relative model performance, we rank the contributions from each of the climate factors. Finally, due to the mechanistic nature of the climate forcing models, we project historical and future nesting trajectories based on available climate data and under different climate change scenarios.

## Results and Discussion

**Figure 2:**
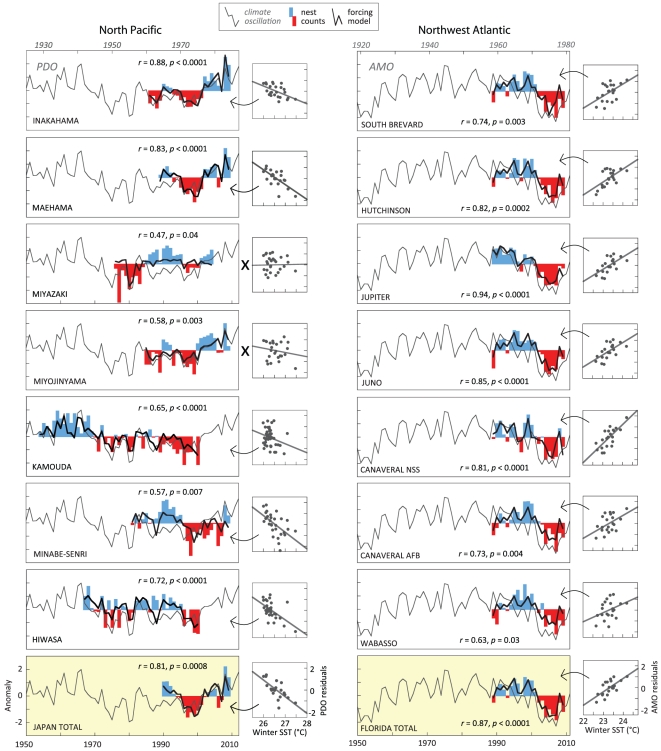
Climate forcing of loggerhead nesting. Nest surveys are positive (blue) and negative (red) annual anomalies plotted against the survey year on the bottom *x* axis. The upper *x* axis is the year of the decadal oscillation (grey line) represented by the PDO and the AMO lagged 25 and 31 years, respectively. Highest-ranked climate forcing model (black line) incorporates the decadal series including (←) or without (x) the winter SST series. Scatter plots display relationships of the winter SST to residuals from the decadal series models alone. Regional total series (yellow panel) accumulates nests from all locations; Japan total provided by the Sea Turtle Association of Japan, Florida total accumulates nests from 15 index nesting beach surveys. Model correlation coefficients and *p*-values are provided.

Loggerhead nesting varies synchronously within regions suggesting that common factors operating over large geographic regions are driving their numbers (Fig. 2). Surveys in Japan and Florida reveal extended periods of high and low abundance, corresponding to well-known fisheries patterns in the same ocean basins [2], [7]. Our climate forcing models account for 18–88% (ave  = 0.60) of the annual variability at the local scale, and 66–77% (ave = 0.71) at the regional scale when nesting data from 1954–2009 are considered (Table S1).

The models capture both annual and decadal time series patterns, including those that have raised concerns with modelers and conservationists. As an example, surveys in Japan indicated a greater than three-fold (linear scale) increase from 2007–2008. While a purely demographic model could not reproduce this trend, our climate forcing model that combines the PDO and winter SST, captures this dramatic increase (Fig. 2). Likewise, the historical decline in Kamouda, Japan from its historical peak in the late 1950s appears to have a climatic component. We note that the Kamouda series does not consistently decline from its first recorded value, but oscillates in concert with the PDO for more than four decades. The declines across Florida from 1998–2007, as well as the increase in the most recent few years, also appear tied to climatic conditions. Importantly, the models perform well locally where most turtles nest. In both ocean basins, nesting abundances at local beaches may vary by an order of magnitude. Since 2000, Inakahama and Maehama represent 36% of all North Pacific nests. Since records began in 1989, South Brevard accounts for 37% of all peninsular Florida nests. The models from these three locations explain on average two–thirds of the annual nesting variability.

**Figure 3:**
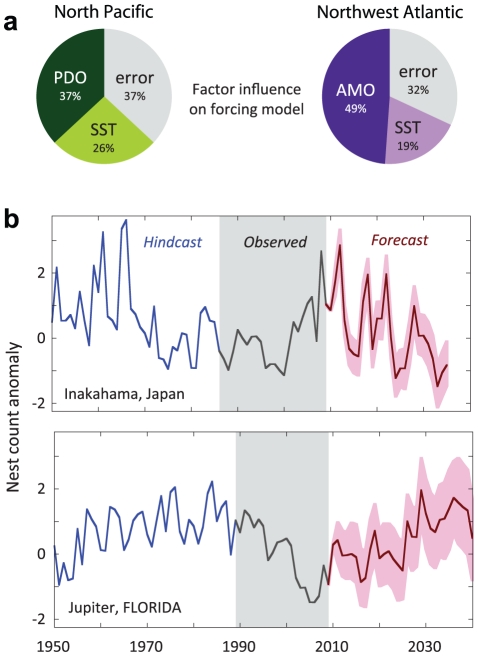
Model factor contributions and population forecasts. (**a**) Pie charts calculate the model contributions from each climate factor, plus error. Percentage values are the sum of squares (SS) improvements from each factor, expressed as an average weighted by the mean annual nest count. (**b**) Nesting anomalies during the historical (blue) and surveyed (black) period, modeled from available climate records. Future forecasts are calculated from observed, lagged oceanographic indices and with stochastic simulations of winter SST (see Methods). Forecast range (pink) and model average (red) are shown. A significant advantage of this climate-based model is the ability to estimate historical and future populations.

The model results also have important demographic implications. As juveniles are the most numerous population segment, we might expect factors regulating their survival could produce a signal detected later in adult surveys. Though Fig. 2 displays the model contributions from each climate factor, Fig. 3a calculates those contributions explicitly and includes the remaining model error. In both populations, the lagged decadal oscillations amount to 70% of the model performance when the results from each series are averaged. Thus, climatic factors in the hatching year are the single most important variable in our forcing models. The influence of juvenile climate factors in explaining nesting abundances may suggest neophyte breeders are a greater proportion of the breeding population than has been recognized. This is consistent with two recent tagging studies in Florida loggerheads which show that most nesting females were first-time breeders [32] and survivorship of recaptured adults was inexplicably low [33]. Though tag loss [34] influences the interpretation of these Florida studies, the research nonetheless corroborates our findings. While our aim in this analysis is a purely climatic population model, future studies might seek to incorporate anthropogenic influences, from direct [35] and indirect harvests [15], for example, or from disease [36]. Time series of such influences, however, can be elusive [37] especially at regional or basin-wide scales. Viewed in context with the present study, factors influencing juvenile recruitment appear to have a dominant role in long-term population trends, something suggested by the renowned sea turtle biologist Archie Carr almost six decades ago [38] and by fisheries biologists beginning a century ago [24].

Age at sexual maturity is an important component in documenting the impact of climate on juvenile recruitment. However, as empirical studies are limited we have no *a priori* global estimate, and we might expect significant geographic variation or demographic stochasticity [39], [40]. In the Florida population, available studies [29], [31] allowed us to fix the lag at 31 years. In the Japan population, without comparable demographic studies, we optimized the lag length using the regional nesting series and fixed all subsequent lags in the Japan population to that value. The optimized lag, in this respect, is a climate-based approximation of age at first breeding. The optimal lag length was calculated to be 25 years for the Japan population (Fig. S1), six years shorter than in Florida. Though demographic information is limited, published records [41] show females breeding in Japan are considerably smaller than those in Florida. As size is positively correlated with age [29], smaller breeding turtles may be younger breeding turtles, other things being equal. In addition, over the past two decades, the number of annual nests in Japan is an order of magnitude below Florida. If this reflects overall population size, lower population densities in the North Pacific may indicate less intraspecific competition, faster growth, and perhaps earlier maturity. Future studies might compare the ocean resources available to each loggerhead population, as has been done elsewhere [42], and examine their influence on population dynamics.

Model relationships calibrated with the empirical survey data are the basis for historical and future nesting projections. Figure 3b uses available climate data to reconstruct historical nesting and available and modeled climate data to forecast future trends. We project forward the lag length, with real PDO and AMO data that have already occurred, but have not yet manifested themselves in nesting populations. Future winter SST is modeled under two scenarios (Fig. S2) based on the historical time series and the IPCC A2 projections [43]. The hindcast projections in Japan approximate the declines observed from 1960–1990 in the longest survey (Kamouda) and predict continued losses extending past 2030. In Florida, unpublished historical surveys (S.J. Epperly personal communication) are consistent with our modeled hindcasts that suggest a population increase from the 1960s through the 1980s. The future outlook in Florida is positive, and shows continued increases in nesting loggerheads to 2040, a result of the AMO signal. We do not project the climate indices beyond the lag periods, but models of the Pacific [44] indicate broad declines. In the North Pacific, projections under anthropogenic climate scenarios indicate a 34% decrease in both the area and primary production of the temperate oceanic biome, which would impact juvenile loggerheads from Japan. We do not have similar projections for the Atlantic and forecasting the oscillation indices themselves has not been possible [26].

The stewardship of marine biodiversity relies on accurate population assessments. While subsistence and commercial harvests preceded large historical declines of marine turtles [13] it is unknown how climatic and anthropogenic forces together regulate the trends of many protected species [45]. Our analysis demonstrates that changes in loggerhead nesting over at least the last several decades are strongly correlated with ocean oscillations. We observed these results in both the Atlantic and Pacific basins, using independent climate series, and when both regional and local population surveys are tested. Our models suggest that oceanographic influences to juvenile recruitment are a major factor, decades later, in breeding populations (Fig. 2) and appear robust to estimate past and future population changes (Fig. 3b).

There are immediate implications for marine turtle population ecology and management. When juvenile and adult life stages are decoupled, population size often has a weak affect on recruitment [4]. Instead, as is the case with many marine fisheries [2], [5], [6], [7], [9], [10], [11] climate forcing seems to dominate population dynamics. By contrast, turtle population assessments often rely on models of adult population growth, entirely independent of environmental factors [31]. Our observation of strong environmental forcing challenges this view. As reptiles with a significant pelagic component, marine turtles may resemble marine fisheries more than terrestrial populations. Future research that addresses the physical and ecological processes that influence pelagic juvenile turtles may provide environmental baselines of population variability. As we demonstrate here, climate models provide new insights into the historical, current, and future populations of marine turtles and provide a mechanistic modeling framework for considering anthropogenic climate change. Our results also add to the ongoing debate on top-down versus bottom-up regulation of wild populations [46], [47], [48], [49].

## Materials and Methods

### Data

Population counts are provided by nest surveys from the Florida Fish and Wildlife Research Institute (FWRI) and published reports, which provide further details [50], [51], [52], [53]. We used linear models of climate variables to estimate ln-transformed [49], [54] nest surveys. PDO series is the calendar year average of the monthly index values, supplied by the Joint Institute for the Study of the Atmosphere and Ocean (JISAO). AMO series is the normalized annual SST anomaly series derived from the Kaplan SST series of the North Atlantic [55], from the NOAA Earth System Research Laboratory (ESRL). SST data are the 2°×2° cell ERSSTv3b series from 1950–1981 and 1°×1° cell OIv2 series from 1981-present [56], from the National Climatic Data Center (NCDC). Japan winter SST is averaged over 8–28°N, 120–128°E [21] during the November–January before the nesting season. Florida winter SST is averaged over 22–38°N, 72–84°W in the previous December [22]. Regional temperature forecasts under the A2 scenario are from the IPCC [43]. We ranked models using the corrected Akaike Information Criterion (AIC_c_) for small samples [57].

### Climate forcing models

Nest counts series were estimated with the following general linear models:



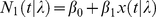
(1)




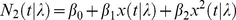
(2)




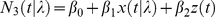
(3)




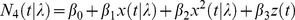
(4)


Here *N_i_* is the annual nesting activity predicted by the *i* ^th^ model. Also *x*(*t*,*λ*) is the oceanographic oscillation index in year *t* lagged by *λ* years and *z*(*t*) is the SST from the previous winter. The numbers *β*_0_, *β*_1_, *β*_2_, *β*_3_, are the fitted model parameters. Nesting activity is defined as the normalized value of the ln-transformed annual nest counts (Kamouda records crawls, not nests). This transformation is consistent with the observed pattern of variability of wild populations [35], [38]. We excluded annual counts <20 (2 of 595 total observations) as such extreme lows skewed the normalization procedure. Model (1) is linear and (2) is a curvilinear relationship to the oceanographic oscillations. Model (3) and (4) add winter SST as a model factor.

We lagged the effect of *x* on *N* as the variability of *x* influences juvenile turtles, only a portion of which breed *λ* years later and are observed nesting. Though we fix this value at 31 years for the Northwest Atlantic population, we have no estimates for the North Pacific population. A recent study [24] hypothesizes 30 years as the global mean time between hatching and first breeding for loggerheads. As we expect this to vary geographically we fit the above models to the observed data for the Japan Total nesting series, separately using lags from 20–40 years. We fix the subsequent local beach series models in Japan to the optimal lag derived from the regional total series.

To rank model performance, we used the corrected Akaike Information Criterion (AIC_c_) for small populations, where:



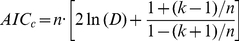
(5)where *k* is the number of model parameters, *n* is the series length and *D* is the mean square deviation between the data and the model [57]. In determining the optimal lag for the Japan Total series, we average the AIC_c_ values from models (1–4) for each lag length and select the lag with the lowest average AIC_c_ (Fig. S1). For the remaining models where the lag length is fixed, the highest-ranked model has the lowest AIC_c_ value of the four tested models. The model contributions from each variable are calculated from their sum of squares.

### Population forecasts

We estimated historical and future nesting in each series using the fitted model relationships. We calculate hindcasts from 1950- using the selected model for each series, including historical oscillation indices and available winter SST data. Because of the lagged influence of the oceanographic indices, we already possess real climate data that has yet to manifest itself in nesting females, *N*. Therefore we forecast nest surveys from 2010 forward, for a period equal to the optimum lag, *λ*. When the models include winter SST, we forecast two scenarios for its change. The first extrapolates from the fitted linear 1950–2010 trend for each region. The second uses an ensemble forecast of multiple Atmosphere-Ocean General Circulation Models (AOGCMs) under the A2 emissions scenario from the IPPC Fourth Assessment Report [43]. The A2 scenario reflects continued global population growth with decentralized ecomonic and technological changes and forecasts more extreme warming than most emission scenarios. The ensemble A2 forecasts show both geographic regions (Fig. S2) have an approximate linear increase of 0.0275°C yr^−1^ during 2010–2040 [43]. To model the uncertainty in the forecast for both scenarios, we added autocorrelated noise based on the power spectrum of noise from the 1950–2010 merged ERSST series [58]. We applied this characteristic noise pattern to the projected annual trends and generated 100 simulations for each scenario. All forecasted SST series were pooled and for each calendar year the forecasted nest abundances is the model average for the ensemble of 200 simulations, essentially, deterministic models within a stochastic shell [59]. We select the upper and lower 2.5% of the simulation forecasts to serve as the upper and lower bounds of the forecast.

## Supporting Information

Figure S1 **Climate lag optimization For North Pacific (Japan) population.** Residual mean square (RMS) values for each model at each lag length. Green lines are two linear models, red lines are two curvilinear models (see Methods). Black line is the average of the two best series. Blue rectangle identifies the optimum lag length according to the data, for the most numerous, the longest, and the regional total series. All three series agree on a 25 year optima.
(EPS)

Figure S2 **Winter sea-surface temperature series.** 1950–1980 data are from the ERSSTv3b series (green) and 1981–2010 are from the OIv2 series (green) provided by NOAA (see Methods). Japan series measures November–January records over 5–28°N, 120–128°E; Florida winter SST measures December records over 22–38°N, 72–84°W – regions shaded green in the inset maps. Future winter SST forecasts are projected linear trends with an added stochastic component derived from the empirical noise. The blue line is forecast according the linear trend of the 1950–2010 data, the red line is according to the Intergovernmental Panel on Climate Change (IPCC) A2 emissions scenario. Both lines are the ensemble average of 100 simulations, given the characteristic noise of the ERSST series.
(EPS)

Table S1 **Details on the surveys and forcing models.** This table describes the nesting beach series and key statistics from the highest-ranked models. DPS is the distinct population segment, a genetic population division made for conservation and management. Optimum lags for each winning model are weighted by local nesting population size in Fig. 3a, separately for Japan and Florida. The *R* and *P* values for forcing models report on goodness-of-fit and statistical significance, respectively. One series (MacArthur, Florida) has a weak statistical correlation.
(DOC)
