# Supplementary material for: Long-Term Climate Forcing in Loggerhead Sea Turtle Nesting
Source: PLoS One. 2011 Apr 27;6(4):e19043. doi: 10.1371/journal.pone.0019043 (PMC3083431; doi:10.1371/journal.pone.0019043)
Supplement: Table S1 — Details on the surveys and forcing models. This table describes the nesting beach series and key statistics from the highest-ranked models. DPS is the distinct population segment, a genetic population division made for conservation and management. Optimum lags for each winning model are weighted by local nesting population size in Fig. 3a, separately for Japan and Florida. The R and P values for forcing models report on goodness-of-fit and statistical significance, respectively. One series (MacArthur, Florida) has a weak statistical correlation. (DOC) [file pone.0019043.s003.doc]

Supplemental Table 1

| **Location** | **Country** | **DPS** | **Observed period** | **Series Length** | **Mean nests yr^-1^** | **Lag length** | **Model number** | ***R*** | ***P* value** |
| --- | --- | --- | --- | --- | --- | --- | --- | --- | --- |
| **Japan Total** | Japan | N Pacific | 1990-2009 | 20 | 4107 | 25 | 3 | 0.812 | 0.001 |
| Inakahama | Japan | N Pacific | 1986-2009 | 24 | 656 | 25 | 4 | 0.883 | < 0.0001 |
| Maehama | Japan | N Pacific | 1989-2009 | 21 | 512 | 25 | 3 | 0.832 | 0.0001 |
| Miyazaki | Japan | N Pacific | 1976-2006 | 29 | 409 | 25 | 2 | 0.469 | < 0.04 |
| Myojinyama-Oida | Japan | N Pacific | 1985-2009 | 25 | 113 | 25 | 1 | 0.576 | 0.003 |
| Omaezaki | Japan | N Pacific | 1973-2000 | 28 | 99 | 25 | 2 | 0.498 | 0.03 |
| Kamouda | Japan | N Pacific | 1954-2000 | 46 | 98 | 25 | 3 | 0.654 | < 0.0001 |
| Minabe-Senri | Japan | N Pacific | 1981-2009 | 29 | 87 | 25 | 3 | 0.566 | 0.007 |
| Hiwasa | Japan | N Pacific | 1967-2000 | 34 | 67 | 25 | 3 | 0.723 | < 0.0001 |
| **Florida Total** | USA | NW Atlantic | 1989-2009 | 21 | 43259 | 31 | 4 | 0.874 | < 0.0001 |
| South Brevard | USA | NW Atlantic | 1989-2009 | 21 | 16035 | 31 | 4 | 0.742 | 0.003 |
| Hutchinson Island | USA | NW Atlantic | 1989-2009 | 21 | 5827 | 31 | 4 | 0.817 | 0.0002 |
| Jupiter Island | USA | NW Atlantic | 1989-2009 | 21 | 3976 | 31 | 3 | 0.936 | < 0.0001 |
| Juno Beach | USA | NW Atlantic | 1989-2009 | 21 | 3828 | 31 | 4 | 0.852 | < 0.0001 |
| Canaveral NSS | USA | NW Atlantic | 1989-2009 | 21 | 3076 | 31 | 3 | 0.810 | < 0.0001 |
| Canaveral AFB | USA | NW Atlantic | 1989-2009 | 21 | 2110 | 31 | 4 | 0.728 | 0.004 |
| Wabasso Beach | USA | NW Atlantic | 1989-2009 | 21 | 1503 | 31 | 4 | 0.632 | 0.03 |
| Patrick AFB | USA | NW Atlantic | 1989-2009 | 21 | 1217 | 31 | 3 | 0.728 | 0.01 |
| Hobe Sound | USA | NW Atlantic | 1989-2009 | 21 | 1215 | 31 | 3 | 0.667 | 0.005 |
| Merritt Island | USA | NW Atlantic | 1989-2009 | 21 | 1046 | 31 | 3 | 0.760 | 0.0004 |
| MacArthur | USA | NW Atlantic | 1989-2009 | 21 | 978 | 31 | 3 | 0.423 | 0.169 |
| Sebastian Inlet | USA | NW Atlantic | 1989-2009 | 21 | 889 | 31 | 3 | 0.657 | 0.006 |
| Boca Raton | USA | NW Atlantic | 1989-2009 | 21 | 599 | 31 | 3 | 0.84 | < 0.0001 |
| Ft. Pierce Inlet | USA | NW Atlantic | 1989-2009 | 21 | 386 | 31 | 2 | 0.592 | 0.02 |
| St. Lucie Inlet | USA | NW Atlantic | 1989-2009 | 21 | 344 | 31 | 1 | 0.549 | 0.01 |
